# An Investigation into the Acute and Subacute Toxicity of Extracts of *Cassipourea flanaganii* Stem Bark In Vivo

**DOI:** 10.3390/plants12122281

**Published:** 2023-06-12

**Authors:** Nomakhosi Mpofana, John Kudakwashe Chipangura, Michael Paulse, Masande Yalo, Nceba Gqaleni, Celenkosini Thembelenkosini Nxumalo, Ncoza Cordelia Dlova, Ahmed A. Hussein, Neil R. Crouch

**Affiliations:** 1 Dermatology Department, Nelson R. Mandela School of Medicine, University of KwaZulu-Natal, Durban 4000, South Africa; dlovan@ukzn.ac.za; 2 Department of Somatology, Durban University of Technology, Durban 4000, South Africa; 3 Research Animal Facility, Faculty of Health Sciences, University of Cape Town, Cape Town 8000, South Africa; john.chipangura@uct.ac.za; 4 Faculty of Health and Wellness Sciences, Cape Peninsula University of Technology, Cape Town 8000, South Africa; paulsemi@cput.ac.za; 5 Department of Chemistry, Cape Peninsula University of Technology, Cape Town 8000, South Africa; yalom@cput.ac.za (M.Y.); mohammedam@cput.ac.za (A.A.H.); 6 Discipline of Traditional Medicine, University of KwaZulu-Natal, Durban 4000, South Africa; gqalenin@ukzn.ac.za; 7 Faculty of Health Sciences, Durban University of Technology, Durban 4000, South Africa; celenkosinin@dut.ac.za; 8 Biodiversity Research and Monitoring Directorate, South African National Biodiversity Institute, P.O. Box 52099, Berea Road, Durban 4007, South Africa; n.crouch@sanbi.org.za; 9 School of Chemistry & Physics, University of KwaZulu-Natal, Durban 4041, South Africa

**Keywords:** *Cassipourea flanaganii*, phytochemical compounds, hypermelanosis, in vivo, toxicity

## Abstract

The conventional use of medicinal plants is in part based on the widespread belief that plant crude extracts are non-toxic. In South Africa, traditional preparations of *Cassipourea flanaganii* used to treat hypermelanosis have accordingly been regarded by many as non-toxic. Whether that is so impacts on the potential of bark extracts to be developed as a commercial drug to treathypermelanosis, given their documented capacity to inhibit tyrosinase activity. Our study investigated the acute and subacute toxicity of the methanol extract of *C. flanaganii* bark in rats. Wistar rats were randomly assigned into different treatment groups. The rats received a daily oral gavage of crude extract for acute and subacute toxicity tests. Haematological, biomechanical, clinical and histopathology examinations were carried out to evaluate the possible toxicity of *C. flanaganii*. The results were subjected to the Student’s *t*-test and ANOVA. For both acute and subacute toxicity, there was no statistical difference between the groups. There were no clinical or behavioral signs of toxicity observed in the rats. No treatment-related gross pathology lesions and no histopathology were observed. The findings of this study demonstrate the absence of acute or subacute toxicity after oral treatment with *C. flanaganii* stem bark extracts in Wistar rats at the levels administered. Chemical profiling of the total extract using LC-MS tentatively identified eleven (11) compounds as the major chemical constituents.

## 1. Introduction

Traditional remedies or medicinal plants are used by 80% of the world’s population living in impoverished countries, as a source of primary health care for preventing and treating diseases [1,2,3,4] such as asthma, diabetes, cardiovascular disorders, cancer, gastrointestinal disorders and skin disorders [5,6]. The use of medicinal plants for disease treatment is mostly motivated by the fact that it is affordable, as well as the widespread belief that plant-based medicines are nontoxic due to their natural origin [7]. However, it is of concern that the most medicinal plants used in conventional healthcare have not undergone their toxicological profile evaluation [8], which makes it important for toxicological and pharmacological evaluation of such plants to protect end users [9]. It has been previously demonstrated that there may be adverse effects and drug interactions caused by natural remedies [1,7,10]. *Cassipourea flanaganii* (Schinz) Alston (Rhizophoraceae) is one of the plants used in traditional medicine in South Africa that has not yet been fully studied for toxicity or safety [8].

*Cassipourea flanaganii* is an endemic South African tree species occurring in forest patches between King William’s Town in the Eastern Cape province and southern KwaZulu-Natal [11]. Its stem bark is used traditionally by African females as a sunscreen, to clear blemishes, improve complexion and lighten the skin tone [10,12]. The stem bark is ground into a powder and mixed with water to form a paste for application to affected areas [12,13], whilst a tincture of the leaves of this species are used for the treatment of wounds and acne [14]. *Cassipourea flanaganii* has demonstrated potential as an alternative to hydroquinone in vitro, as it contains tyrosinase inhibitors and anti-inflammatory properties [13,15]. Tyrosinase inhibitors are extensively used as complexion modifiers in the cosmetic industry [16]. The use of plant extracts and phyto-constituents derived from them has a promising future in the treatment of hyperpigmentation disorders [16].

Studies dedicated to elucidating the phyto-constituents of *C. flanaganii* are limited. Langat et al. reported one new compound, along with fourteen known compounds from chloroform and methanol stem bark extracts of *C. flanaganii* [13]. The new compound was characterised and identified as ent-atis-16-en-19-al and the known compounds as docosyl ferulate, *ent*-atis-16-en-19-oic acid, β-amyrin, *ent*-atis-16-en-19-ol, lupeol, *ent*-kaur-16-en-19-oic acid, *ent*-kaur-16-en-19-al, lynoside, *ent*-manoyl oxide, lichenxanthone, guinesine A, guinesine B and guinesine C. Methanol extracts of *C*. *flanaganii* have been reported to exhibit both anti-tyrosinase and anti-inflammatory activities [13,17]. *C. flanaganii* acetone, as well as water extracts have already been demonstrated to have the ability to function as two distinct inhibitors of the COX-2 enzymes and 15-LOX [15].

Although the stem bark of *C. flanaganii* is used traditionally, very little is known about the possible systemic toxicity. We accordingly further investigate toxicity using an animal in vivo model, to support the evaluation of the risk/therapeutic benefit ratio, and so to protect users from possible systemic toxicity. A key issue regarding the use and promotion of most medicinal plants relates to whether the benefit–risk balance is appropriate and efficient for monitoring safety [10,18]. It is known that mercury-containing products that are used as skin lighteners can be absorbed through the skin and may cause end-organ damage. In addition, topical steroids have also been shown to cause suppression of the hypothalamic–pituitary–adrenal (HPA) axis after prolonged use [19,20]. Hence, it is always important to find out if any topical treatment can cause systemic side effects by administering it orally to observe any systemic end-organ uptake.

In this context, the current study focuses on assessing the safety profile of the *C. flanaganii* plant extract through the consideration of acute and subacute toxicity in rats. We evaluated the effect of the extract on different organs and also the clinical signs associated with toxicity. Toxicity testing in animals is an important part of the drug development process, as it identifies adverse effects of a substance on different organ systems, estimating its lethal dose, and further informs the initial safety guidelines for human exposure. These data guide the design of human clinical trials to help secure the safety of volunteers and patients who participate [21]. The widespread use of *C. flanaganii* as a complexion modifier and skin lightening product has warranted further safety and efficacy research. The ensuant findings should add a new dimension to the search for the safe and effective tyrosinase inhibitors derived from medicinal plants.

Although the stem bark of *C. flanaganii* is used traditionally, very little is known about the possible systemic toxicity. Using an animal in vivo model, we accordingly further investigate the toxicity in relation to real therapeutic effects, to evaluate the risk/benefit ratio to protect users from possible systemic toxicity. A key issue regarding the use and promotion of most medicinal plants relates to whether the benefit–risk balance is appropriate and efficient for monitoring safety [10,18].

In toxicity studies, haematological parameter tests reveal the extent to which foreign compounds, including plant extracts, have a negative effect on animal blood constituents, and can also explain how chemical compounds/plant extracts affect the blood. Haematological analyses are also relevant for risk assessment, since changes in the blood system can be used to predict toxicity in humans when data from animal studies are translated [22,23,24].

A reduced platelet count (RBC) may indicate that the blood clotting process may be hampered. The red cell distribution width (RDW) is a measurement based on red blood cell distribution curves produced by automated haematology analyses, which can be utilized for assessing RBC size variation within a blood sample [2,25]. The RDW, in conjunction with the indices of the mean corpuscular volume (MCV), mean corpuscular haemoglobin (MCH) and mean corpuscular haemoglobin concentration (MCHC), is used to describe a population of RBCs [2,25]. Biochemical parameters include total serum protein, alkaline phosphate (ALP), alanine aminotransferase (ALT), aspartate aminotransferase (AST), albumin, total bilirubin, urea, creatine, low-density lipoprotein cholesterol (LDL-C), high-density lipoprotein cholesterol (HDL-C) and triglyceride [26,27] and may indicate any toxicity related to the liver. Liver cell damage in toxicity studies is indicated by the increase in AST and ALT, the most specific marker of liver cell damage [26,27].

This study was carried out to investigate the acute and subacute toxicity of the methanol extract of *C. flanaganii* bark in Wistar rats. The data generated from the current animal model toxicity study could be used for toxicity classification, and assessing the safety profile of the plant extract could inform clinical trials, as well as the possible formulation and commercialization of *C. flanaganii* as a tyrosinase inhibitor.

## 2. Results

The results of this study are reported according to the ARRIVE Guidelines 2.0 for the publishing of animal experiments (Supplementary Materials File S1). The guidelines are supported by numerous journals and are intended to improve and strengthen the quality of animal experiment reporting [28,29]. The administration of *C. flanaganii* methanol stem bark extracts at the doses of 50, 300 and 2000 mg/kg body weight to both male and female Wistar rats during the 14-day repeat acute toxicity study exhibited variable effects on the biochemical parameters. Similarly, a comparable outcome was observed during the 28-day subacute toxicity study period where 100, 500 and 1000 mg/kg doses of *C. flanaganii* methanol stem bark extracts had variable effects within the 14-day repeat acute study (Table 1 and Table 2), as well as subacute study. For the 28-day subacute study, changes were noticed on both biochemical, as well as haematological parameters.

### 2.1. Chemical Profile

**Figure 1:**
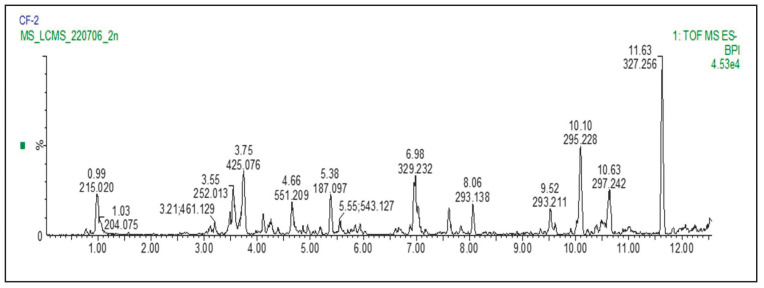
LC-MS chromatogram of *C*. *flanaganii* extract in the negative mode.

**Figure 2:**
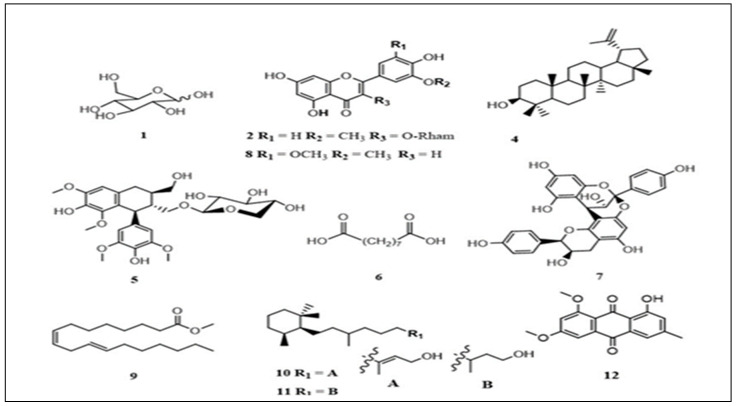
Chemical constituents identified in *C. flanaganii*.

An LC-MS chromatogram of the extract of the stem bark of *C*. *flanaganii* plant species (Figure 1) showed 13 major peaks, indicating the presence of 13 major constituents of the extracts (Figure 2).

The tentative identification of the constituents (compounds) was based on the retention time with authentic samples (compounds **2** and **8**) and mass fragmentation pattern with the help of the available mass spectra *m*/*z* databases such as Mass Bank of Europe and the National Library of Medicine, and related literature reviews. The chromatogram results for *C. flanaganii* revealed the presence of hexose (could be glucose) (215.033 *m*/*z*), isorhamnetin-3-*O*-rhamnoside (461.129), lupeol (425.075), lynoside (551.202), azelaic acid (187.097), mahuannin B (543.127), tricin (329.232), methyl linoleate (293.211), cassipouriol (293.211), decahydroretinol (295.228) and emodin 6,8-dimethyl ether (297.242) (Table 1).

### 2.2. Morphological Alteration

No clinical or behavioural signs (e.g., piloerection, changes in locomotor activity or changes in food and water intake) of toxicity were observed in the rats after the single oral gavage with the plant extract, both at the highest and lowest doses for both the acute and subacute studies. For the acute phase, all the rats survived until day 14, with the exception of one rat that died a day after dosing. Upon an examination of the carcasses, no treatment-related pathological changed were observed. The experiment was repeated once, with none of the rats dying. For the subacute phase, all the rats survived until day 28. Additionally, there was no evidence of tonic-clonic movements, stereotypies such as excessive grooming and repeated circling, or any odd behaviour such as backward walking or self-mutilation. Thus, motor activity was unaltered by the *C. flanaganii* crude extract administration. The postmortem examination of the carcasses on the last day of the experiment revealed no treatment-related gross pathological changes, as there were no clinical signs observed nor any macroscopic changes recorded at necropsy. Clinically, no treatment-related microscopic pathological changes were observed.

### 2.3. Fourteen-Day Acute Toxicity

In phase one (Table 2) of the acute toxicity study, the concentrations of total protein (g/L), albumin (g/L), globulin (g/L) and ALT (IU/L) were increased relative to the control group, urea was increased in the 50 mg/kg group, albumin also increased in the 300 mg/kg group. From the 2000 mg/kg, the total protein, albumin, globulin and alb/glob ration was altered. Similarly, during the repeat phase (Table 3), the total protein, globulin and AST concentrations were increased from the control group. From the 500 mg/kg group, only the total protein was altered, and lastly a noteworthy significant difference was evident from the 2000 mg/kg group as total protein, albumin, globulin and ALK were increased.

### 2.4. Fourteen-Day Acute Repeat Toxicity Results

For the 14-day acute study (Table 2), the total protein (control group, 2000 mg/kg), albumin (control and 300 mg/kg, 2000 mg/mg), ALT (control) and urea (50 mg/kg), and the alb/glob ratio (2000 mg/kg) were shown to be significant. Similarly, for the repeat phase (Table 3), it was observed that the differences in the total protein (control and 50 mg/kg, 2000 mg/kg), globulin (control and 2000 mg/kg), AST (control group) and ALK (2000 mg/kg) were significant. These significant findings are exceptional, as all other assays were not significant at the 5% level, i.e., indicative of non-toxicity of the crude plant extract.

#### Subacute Biochemical Analyses

The independent Student’s *t*-test for the mean comparison between the “baseline” chemistry for each concentration (the control, 100, 500 and 1000 mg/kg) compared to their respective end “values” showed no statistical difference between the baseline and the end values (Table 4). However, the levels of urea were altered in the 500 mg/kg group, with the baseline of 6.47 ± 0.80 and an end value of 9.25 ± 0.56, as well as in the control group, as indicated by the baseline (6.26 ± 0.44) and end value (9.14 ± 0.73). Similarly, ALK levels were altered throughout (Table 3) within the 100 mg/kg group (baseline 255.20 ± 72.65: end 136.70 ± 51.53), 500 mg/kg (baseline 252.40 ± 86.03: end 143.40 ± 46.25), 1000 mg/kg (baseline 239.10 ± 61.87: end 136.80 ± 49.24), as well as the control group (baseline 288.50 ± 58.88: end 193.40 ± 45.39). In contrast, the single-factor ANOVA analysis (Table 5) showed no statistical (NS) “between-group” differences when comparing the 100 mg/g, 500 mg/kg, 1000 mg/kg and the control for the biochemical analyses. The implication of this finding indicates non-toxicity of the plant extract.

### 2.5. Haematological Analyses

For the 28-day acute study, the independent Student’s *t*-test for the mean comparison between the “Baseline” full blood count and chemistry for each concentration, viz., the control, 100 mg/kg, 500 mg/kg and 1000 mg/kg compared to their respective “End” values showed no statistical differences between the “Baseline” and the “End” values. This is shown as NS in Table 6 and Table 7. There are, however, three anomalies (Table 6).

- White cell count (500 mg/kg) at the Baseline (4.50 ± 2.06) and End (6.77 ± 2.28), the difference being statistically significant (SIG);
- Monocytes ABS (500 mg/kg) at the Baseline (0.07 ± 0.04) and End (0.16 ± 0.60), the difference being statistically significant (SIG); and
- Monocytes ABS (Control) at the Baseline (0.08 ± 0.03) and End (0.16 ± 0.05), the difference being statistically significant (SIG).

The single-factor ANOVA tests (Table 7) revealed no significant (NS) “between-group” differences when comparing the 100 mg/g, 500 mg/kg, 1000 mg/kg and the control for the full blood count.

### 2.6. Distribution of Organ Weights

The Shapiro–Wilk test for normality was performed (Table 8) to assess whether the organs recovered from the euthanized rats at the end of the study were normally distributed.

**Figure 3:**
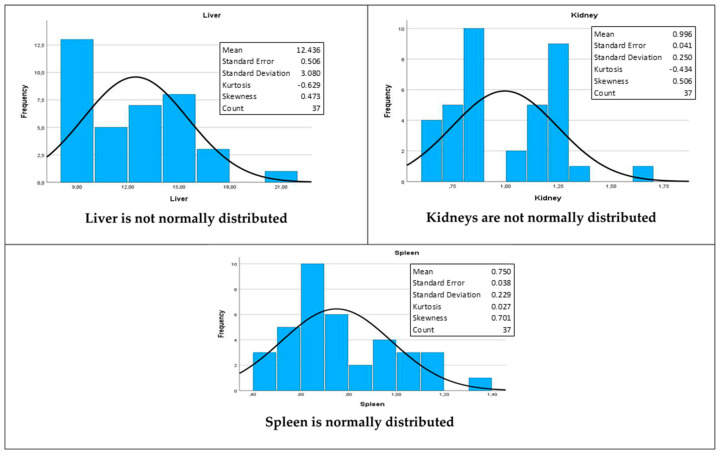
The effect of *C. flanaganii* extracts on indicator organs of Wistar rat organ weight histograms with superimposed normal density curves, indicated together with descriptive statistics.

Given that the null hypothesis for this test [38] is that the variable in question, i.e., organ weights, is normally distributed, the livers (W = 0.931, *p* = 0.023) and kidneys (W = 0.913, *p* = 0.007) were found to not be normally distributed at a 5% level of significance. However, the spleen (W = 0.948, *p* = 0.081) was found to be normally distributed. Their respective histograms, with superimposed normal density curves, are shown in Figure 3 below.

### 2.7. Changes in Weight over Time

**Figure 4:**
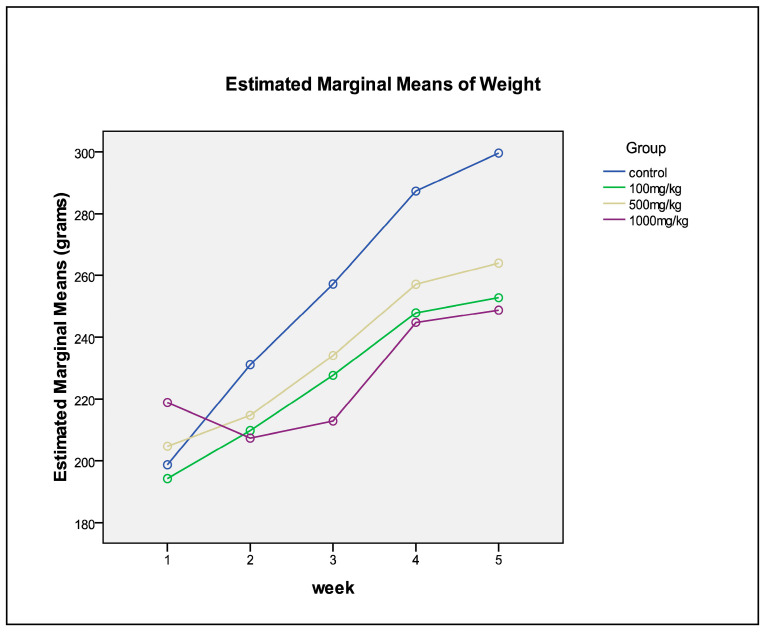
Weight gain in Wistar rats related to the treatment with *C. flanaganii* extracts.

Analysis shows that the change in weight across the weeks differs depending on which experimental group the rats are in. The interaction between the group and time is significant (*p* = 0.005). With the exception of the 1000 mg/kg group, which reflected a decline between weeks 1 and 2, all the groups showed an increase in weight. This is reflected in Figure 4 below.

## 3. Discussion

The study aimed to evaluate acute and subacute toxicity of *C. flanaganii* in Wistar rats. To determine the potential human health hazards caused by the intrinsically harmful effects of the chemical compounds/plant extracts, animal toxicology studies are often carried out. These adverse effects may induce considerable alterations in enzyme and metabolic product levels, as well as normal organ function and histomorphology [24]. The use of medicinal plants for disease treatment is typically motivated by the belief that they are low in toxicity due to their natural origin. In contrast to the popular view, scientific evidence has demonstrated that some herbal bioactive agents have negative effects that are attributed to plant secondary metabolites [18,23,40]. The common use of *C. flanaganii* by people in rural communities suggests that they perceive it to be safe for use because it does not show signs of short-term side effects. However, chronic illnesses such as cancer, and kidney and liver damage could be associated with the overuse of some medicinal plants. This dearth of safety data could lead to adverse effects on the users, hence this study investigated the effects of *C. flanaganii* methanol extract in vivo.

Any new drug or pharmaceutical should be studied for safety and toxicity in appropriate animal models before being provided to human volunteers and patients, as stated by the worldwide opinion and legislation pertaining to human health [41]. The toxicity studies often measure risk by taking into consideration the likelihood of being exposed to a specific danger at various levels [42]. Acute toxicity tests are commonly used to provide preliminary information on a material’s toxic nature and determine the minimum lethal or maximum non-lethal dose, and accurately elucidate the toxicity of medicinal plants [38]. Toxicity information obtained from these studies is useful when dealing with cases of accidental ingestion of a large amount of the material, as it can identify the possible target organs that should be scrutinized; it is also recommended that special testing that should be performed in repeated-dose toxicity tests, and the selection of doses for short-term and sub-chronic toxicity tests [42]. Occasionally, the information from acute toxicity studies is used to define doses for other research, and in such circumstances, the pathological examination is usually confined to macroscopic observations so that target organs could possibly be identified [41].

The LC-MS analysis resulted in 13 major peaks, and 11 of them were identified tentatively, as given in Table 1. The majority of the compounds were phenolic in nature, with some of them reported from similar species such as cassipourol, which has been previously identified in *C. madagascariensis* with other lupeol derivatives [38]. Some of the identified compounds, such as lupeol [32,33], lynoside [13], cassipourol, decahydroretinol [38] and azelaic acid [34], have been reported to have low to no cytotoxicity.

In this study, during phase one of the acute toxicity study, the concentrations of total protein (g/L), albumin (g/L), globulin (g/L) and ALT (IU/L) were increased from the control group, urea was increased in the 50 mg/kg group, albumin also increased in the 300 mg/kg group, from the 2000 mg/kg, the total protein, albumin, globulin and the alb/glob ratio was altered. Similarly, during the repeat phase, the total protein, globulin and AST concentrations were increased from the control group. In addition, it was observed that from the 500 mg/kg group, only the total protein was altered, and lastly, another significant difference was evident from the 2000 mg/kg group, as the total protein, albumin, globulin and ALK were increased across all groups. These altered blood levels across the groups at different concentrations are not a true reflection of the effects of the plant extract. It is noteworthy that the blood levels were altered in the control group too, which only received distilled water. Therefore, it is plausible to conclude that before the study commenced, the rats had an altered total protein, albumin and ALT. Furthermore, the pathological examination of the carcasses revealed no pathological gross alteration of the vital organs associated with the extract.

For the 28-day haematological analysis, the effects of the *C. flanaganii* extract in this study shows that the white cell count and monocyte ABS are significantly different (Table 6), however they are still within normal ranges for rats [43]. Additionally, the white cell count and monocyte ABS showed that there is a significant difference even in the control group. It has previously been suggested that toxic plants have no immediate impact on white blood cells [27,44]. The current results are inconclusive as the control group, where distilled water was administered, also showed a significant difference. It may be that the rats were anaemic or had some form of disease or invading organisms which affected their white blood cells and ABS monocytes before the study began, as their main function is to defend against foreign invading organisms, hence they seem to have been compromised.

Biochemical analyses showed altered levels for both urea (the 500 mg/kg and the control groups) and ALK (across all study groups) (Table 4). The ALK alterations could indicate problems relating to the liver or bones, while urea may be an indication that the kidneys are not working well. In support of the altered levels, the Shapiro–Wilk test for normality found the liver and kidneys not to be normally distributed (Figure 3), as illustrated by the histograms with superimposed normal density curves (Figure 3). Similarly, to the haematology results, these altered biochemical values may be incidental findings and may not necessary be attributed to the toxicity of the extract, as they are observed even in the control group. It may be that the rats had liver and kidney problems before the study. Having said that, the possibility of both the liver and kidney problem is ruled out, since the pathologist did not find any liver pathology upon histopathology. Perhaps the problem was not severe enough to reflect in the histopathology.

All groups showed an increase in weight, with the exception of the 1000 mg/kg group, which reflected a decline between weeks 1 and 2 (Figure 4). The organ–body weight ratio measures swelling, atrophy or hypertrophy [24]. A rise in this measure may indicate inflammation, whereas a reduction may indicate cellular constriction. Serum proteins, such as albumin and globulin, are crucial indicators of the liver’s secretory ability/functional capacity. Renal function indicators such as serum electrolytes, urea, creatinine and uric acid, could be utilized to evaluate the animal nephron functioning of animals, of which none of these parameters showed alteration, with the exception of urea (Table 4). The primary reason for weight gain in this study could be that the animals were gaining weight normally, as they were only a few weeks old when they were enrolled in the study. Pathological examination showed no evidence of the increased body weight being associated with the toxicity of the plant extract. Although organ weight showed an abnormal distribution of the liver and kidneys, it may not be associated with weight gain, as there was no pathological evidence of toxicity associated with the increased weight at the completion of the study period. The level of toxicity of a drug is measured by the lethality and degeneration of vital organ tissues or cells, particularly the liver and kidney tissues or cells [24]. In this study, a daily oral administration of 100 mg/kg, 500 mg/kg and 1000 mg/kg via an oral gavage to male and female rats was not associated with any microscopic changes in any of the liver, spleen or kidney tissues at day 28.

The results of this study demonstrated no significant changes in the concentrations of major lipids such as LDL-C, HDL-C, cholesterol and triacylglycerol. Usually, these changes in lipids could provide significant details on the susceptibility of the hearts of animals to atherosclerosis, as well as coronary heart disease. Lipolysis, plasma cholesterol carrier and atherosclerotic propensity are all associated with triacylglycerol, LDL-C and HDL-C, respectively. The treatment with *C. flanaganii* extracts over the period did not affect the state of the Wistar rat livers, kidneys or spleens, as evidenced by the histopathology. This paper demonstrates that different doses of *C. flanaganii* administered orally over 28 days showed no systemic toxicity, as no body weight loss or weight changes in target organs for metabolism and excretion (liver and kidneys), including the spleen, were observed. Although there were alterations in haematology and biochemistry, the alterations cannot be attributed to the toxicity of the plant extract, as changes were observed even in the control group. From the results of our study, it is plausible to conclude that *C. flanaganii* has low acute or subacute toxicity in the rat blood system.

The results of this study support the further development of an alternative treatment for hypermelanosis disorders to the stem bark of *C. flanaganii*. *Cassipourea flanaganii* is threatened with extinction, as it is highly sought after by communities for traditional remedies, and less than 2500 individuals remain in the wild. As it is a stem bark that is harvested destructively, this results in death through ring barking of individual trees. Should constituents from this species ultimately be shown to be commercialisable, it is recommended that large-scale community-based cultivation be motivated, as residual wild plant stocks are insufficient to meet commercial needs. Community awareness and education on methods of preserving the plant should be established. We consider that, given the high unemployment and extensive poverty in South Africa, indigenous communities should be assisted in the commercialisation and related job creation linked to the economic development of the country’s flora.

## 4. Materials and Methods

The study was approved by the University of Cape Town, Faculty of Health Science Animal Ethics Committee (protocol number: 020_009), as well as the University of KwaZulu-Natal Animal Ethics Research Committee (AREC/034/019D). All animal experiments were conducted following the relevant guidelines for the care and use of animals for scientific purposes in South Africa (SANS 10386).

### 4.1. Plant Material

The crude stem bark of *C. flanaganii* was collected from Pirie forests, Qonce, in the Eastern Cape. The leaf material of the plants sampled were identified by one of the authors, Professor Neil Crouch, a botanist based at the South African National Biodiversity Institute (SANBI). The voucher specimen (NH0151951-0) was deposited at the KwaZulu-Natal Herbarium (NH) in Durban. A permit to harvest the bark material was obtained by the first author from the Department of, Forestry, Fisheries and the Environment (DFFE) (12/11/1/7A (JD)).

### 4.2. Preparation of Extract

For 4 weeks, the bark was dried in shade, prior to being pulverized. The pulverized crude stem bark (918.3 g) was soaked in methanol (1.5 L) at room temperature (±25 °C) for 48 h and then filtered through Whatman Grade 42 paper (Whatman plc; Maidstone, UK) to obtain methanolic filtrates. From the filtrate, the methanol solvent was entirely evaporated using a rotary evaporator that operates at a low pressure of less than 40 °C, after which it was dried under a constant supply of cool air. The evaporation produced 36.8 g (4.01%) of methanolic extracts. The extracts were stored at 4 °C before in vivo studies. Methanol was the solvent of choice in order to maximize and obtain extracts with the highest biological activity [45].

### 4.3. LC-MS Analysis

Liquid chromatography mass spectrometry (LCMS) analysis was carried out using a Waters Synapt G2Quadrupole time-of-flight (QTOF) mass spectrometer (MS), connected to a Waters Acquity ultra-performance liquid chromatography (UPLC) instrument (Waters, Milford, MA, USA). Electrospray ionization was used in the negative mode with a cone voltage of 15 V, desolvation temperature of 275 °C, desolvation gas at 650 L/h and the rest of the MS settings optimized for the best resolution and sensitivity.

### 4.4. Animal Housing and Care

Wistar rats (+/−200 g) were supplied by the University of Cape Town Research Animal Facility, and were housed in the BSL-1 Experimental Rat Unit in Type IV conventional rodent cages, with wood shavings provided as bedding. The rodent food pellets and water were provided *ad libitum*. Red Perspex tubes and gnawing blocks were provided as enrichment. Before experimental procedures, all rats were acclimated for at least 7 days, during which they were exposed to human handling and experimental environmental conditions (room temperature: 20–24 °C; humidity: 55 ± 15%; lighting cycle: 12 h light/12 h dark).

### 4.5. Experimental Procedure

#### 4.5.1. The 14-Day Acute Toxicity Study

The 14-day acute toxicity study was repeated twice. For each study period, 12 female rats were randomly assigned to 4 treatment groups (control (C); 50; 300; 2000 mg/kg body weight), comprising 3 rats each. A one-off pre-dose blood sample was collected. The rats were fasted for 4 h before one-off dosing, and weighed before the test compound was administered (oral gavage), with each rat receiving 10 mL/kg (body weight of rat) volume of the plant extract at most. The doses were based on the OECD guidelines for acute and subacute toxicity testing, and were calculated based on the weight of the rats. For example, the plant extract was dissolved in 70% methanol to make a 400 mg/mL syrup/solution. A 250 g rat was dosed with 0.63 mL at 1000 mg/kg, with each rat receiving 10 mL/kg (body weight of rat) volume of the plant extract at most. Thus, a single oral dose (LD) of 50 mg/kg bw, medium dose (MD) of 300 mg/kg bw and a high dose (HD) of 2000 mg/kg bw, the *C. flanaganii* extracts were made into aqueous homogeneous suspensions. The control group (Group 1) was administered drinking water once. Group 2 (low dose), group 3 (medium dose) and group 4 (high dose) were administered the plant extract as a single administration, with doses indicated previously. After the plant extract administration, food was withheld for 30 min.

#### 4.5.2. The 28-Day Subacute Toxicity Study

Both male and female rats were randomly assigned 4 treatment groups (control; 100; 500; 1000 mg/kg body weight), comprising 10 rats each (5 males and 5 females). A one-off pre-dose blood sample was collected. The rats were weighed once a week and the weekly weight was used to calculate the amount of test compound to be administered for that week via oral gavage. The daily oral dose (LD) of 100 mg/kg bw, medium dose (MD) of 500 mg/kg bw and a high dose (HD) of 1000 mg/kg bw *C. flanaganii* extracts were made into aqueous homogeneous suspensions. The control group (group 1) was administered drinking water. Group 2 (low dose), group 3 (medium dose) and group 4 (high dose) were administered the plant extract at a daily administration of test doses for a period of 28 days.

### 4.6. Clinical Observations

For both the acute and subacute toxicity studies, the animals were observed individually after dosing at least once during the first 30 min, periodically during the first 24 h, with special attention given during the first 4 h, and once a day thereafter. Clinical observations were carried out by a qualified veterinary scientist (Dr John Chipangura). All observations were carefully recorded, with separate files kept for each rat. The observations included changes in the skin, fur, eyes, mucous membranes, respiratory, circulatory, autonomic and central nervous systems, and behavioural patterns. Attention was also directed to observations of tremors, convulsions, salivation, diarrhoea and lethargy. Animals that reached humane endpoints (severe pain or enduring signs of severe distress) were humanely euthanized. At the end of the study, the rats were euthanized with an anaesthetic overdose of isoflurane.

### 4.7. Sample Collection

For both the acute and subacute toxicity studies, baseline blood samples were collected from the tail vein into EDTA and serum separator tubes for haematological and biochemical analyses, respectively. The blood collected in serum separator tubes was centrifuged at 1500 rpm for 10 min and serum was collected for subsequent biochemical analysis. At the end of the experiment, the rats were anesthetized with isoflurane, and blood samples were collected via cardiac puncture, followed by a complete post-mortem examination. The kidney, liver, and spleen were collected for histopathological evaluation.

### 4.8. Hematological Indices

The haematological analysis was carried out at PathCare Veterinary Laboratories (Cape Town, South Africa), using a haematology analyser. The haematological parameters analysed included RBCs, Hb, PCV, MCV, MCH, MCHC, and RCDW, WBC, neutrophils, monocytes, lymphocytes, eosinophils, basophils and platelets.

### 4.9. Biochemical Analysis

Biochemical analyses were performed using the serum centrifuged from the blood collected in serum separator tubes. ALT, AST, ALP, urea, uric acid, creatinine, total bilirubin, total protein, sodium, potassium, chloride, magnesium, calcium, inorganic phosphorus, albumin, globulin, LDL-C (mg/dL), HDL-C, triglycerides and cholesterol levels were analysed in rat serum using standard methods on an automated chemical analyser. Biomechanical analysis was carried out at PathCare Veterinary Laboratories, Cape Town, South Africa.

### 4.10. Histopathology

At the completion of the study period, after the animals were euthanised, the liver, kidney and spleen were collected and stored in neutral-buffered 10% formalin for histopathological examination. The samples of tissue were dehydrated in alcohol, cleaned with xylene and embedded in paraffin. They were later sectioned at a thickness of 5 µm, ensuring that the section contains only a single layer of cells, and later stained with haematoxylin and eosin. The tissue sample examination was carried out at PathCare Veterinary Laboratories, Cape Town. Under a light microscope, general structural changes, degenerative changes, necrosis and signs of inflammation were studied.

### 4.11. Statistical Analyses

Statistical Package for Social Sciences (SPSS) version 28.0 was used to statistically analyze the data. The 95% confidence interval of the calculated data was expressed as the mean ± critical t-value × standard error. Two independent statistical tests were performed: the independent Student’s *t*-test for paired data, and the analysis of variance (ANOVA) test was utilized to identify statistical difference “between the groups”.

1. For paired data, the independent Student’s *t*-test was used to determine whether there was a statistical difference between the “Baseline” and its corresponding “End” value. The “Baseline” full blood count and chemistry for each concentration, viz., the control, 100 mg/kg, 500 mg/kg and 1000 mg/kg were compared to their respective “End” values. Similarly, this test was also conducted for the acute toxicity studies at each different level of toxicity, i.e., the control, 50 mg/kg, 300 mg/kg and 2000 mg/kg. Statistical significance was defined as a probability value of *p* < 0.05 and designated as SIG in subsequent tables. Where this test yielded an insignificant finding, it was designated as NS. N/A denoted a situation where the test could not be conducted, e.g., no observable variation in the observed data.
2. In addition, analysis of variance (ANOVA) tests were utilised to identify statistical differences “between the groups”, i.e., using single-factor ANOVA tests to identify statistical differences between all the “End” values. The comparing groups were the control, 100 mg/kg, 500 mg/kg and 1000 mg/kg concentration levels. Once again, the same denotation was applied, i.e., SIG, NS and N/A.

In addition, the animals’ final body weights were recorded over the four-week period and discussed for observed changes against the initial baseline weight. Organ weight was also tested for normality using the Shapiro–Wilk normality test.

## 5. Conclusions

The study evaluated the acute and subacute toxicity of *C. flanaganii* methanolic stem bark extracts in rats. During the14-day acute and 28-day subacute toxicity study period, the findings of this study demonstrate the absence of acute or subacute toxicity after oral gavage with *C. flanaganii* methanolic bark extracts in Wistar rats. A histological examination of the vital organs showed no gross alterations, suggesting no morphological disturbances. Thus, no toxicity was observed at the levels administered. Clinical trials should also be conducted to complete the safety assessment of *C. flanaganii* use in humans. The findings of this study inform the possible commercialisation of *C. flanaganii* as a tyrosinase inhibitor derived from a medicinal plant, in support of melasma treatment. Efforts to conserve this species through large-scale community-based cultivation should be motivated as part of a formal Biodiversity Management Plan for this species.

## Figures and Tables

**Table 1:** Phytochemical compounds identified in the crude extract of *C*. *flanaganii* via LC-ES-TOF-MS analysis in the negative mode.

Peak	Proposed Compound	*m*/*z*	t_R_ (min)	[M − H]^−^	Molecular Formula	Refs.
1	Hexose	215.033	0.99	[M + Cl]^−^	C_6_H_12_O_6_	[30]
2	Isorhamnetin-3-*O*-rhamnoside	461.129	3.21	[M − H]^−^	C_22_H_22_O_11_	[31]
3	Unknown	252.014	3.55	[M − H]^−^	Unknown	-
4	Lupeol	425.075	3.75	[M − H]^−^	C_30_H_50_O	[32,33]
5	Lynoside	551.202	4.66	[M − H]^−^	C_27_H_36_O_12_	[13]
6	Azelaic acid	187.097	5.38	[M − H]^−^	C_9_H_16_O_4_	[34]
7	Mahuannin B	543.127	5.55	[M − H]^−^	C_30_H_24_O_10_	[35]
8	Tricin	329.232	6.98	[M − H]^−^	C_17_H_14_O_7_	[36]
9	Methyl linoleate	293.138	8.06	[M − H]^−^	C_19_H_34_O_2_	[37]
10	Cassipourol	293.211	9.53	[M − H]^−^	C_20_H_38_O	[38]
11	Decahydroretinol	295.228	10.10	[M − H]^−^	C_20_H_40_O	[38]
12	Emodin 6,8-dimethyl ether	297.242	10.63	[M − H]^−^	C_17_H_14_O_5_	[39]
13	Unknown	327.256	11.63	[M − H]^−^	Unknown	-

**Table 2:** Student’s t-test comparing baseline to the end-of-study results at various levels of toxicity for phase one: 14-day acute toxicity results.

Toxicity Test	Control Baseline			End Value			*p*-Value	Decision	50 mg/kg Baseline			End Value			*p*-Value	Decision
Urea	7.90	±	1.79	6.67	±	0.57	0.118	NS	8.10	±	0.43	6.33	±	0.57	0.005	SIG
LDL Cholesterol	N/A			N/A				N/A	N/A			N/A				N/A
Triglyceride	0.66	±	0.42	0.65	±	0.54	0.716	NS	0.73	±	0.74	1.16	±	0.73	0.290	NS
Total protein (g/L)	68.00	±	8.96	63.00	±	6.57	0.013	SIG	70.67	±	11.20	64.67	±	5.17	0.188	NS
Albumin (g/L)	35.00	±	4.97	32.00	±	2.48	0.035	SIG	36.00	±	4.97	32.67	±	2.87	0.130	NS
Globulin (g/L)	33.00		4.30	31.00	±	4.30	N/A	N/A	34.67	±	6.25	32.00	±	2.48	0.270	NS
Alb/Glob ratio	1.07	±	0.14	1.03	±	0.14	0.423	NS	1.03	±	0.14	1.00	±	N/A	0.423	NS
Total Bilirubin	20.0	±	N/A	2.00	±	N/A		N/A	2.33	±	1.43	2.00	±	N/A	0.423	NS
ALK (IU/L)	107.00	±	36.08	106.67	±	36.62	0.979	NS	103.33	±	72.10	84.67	±	33.08	0.333	NS
ALT (IU/L)	42.67	±	11.20	37.33	±	11.20	0.004	SIG	42.00	±	22.77	35.00	±	15.51	0.379	NS
AST (IU/L)	102.67	±	11.20	88.00	±	10.83	0.065	NS	107.33	±	42.33	68.67	±	10.04	0.054	NS
**Toxicity Test**	**300 mg/kg Baseline**			**End**			***p*-Value**	**Decision**	**2000 mg/kg Baseline**			**End**			***p*-Value**	**Decision**
Urea	8.37	±	1.88	7.20	±	1.08	0.194	NS	7.17	±	1.37	7.15	±	0.87	0.939	NS
LDL Cholesterol	N/A			N/A				N/A	N/A			N/A				N/A
Triglyceride	0.77	±	0.68	0.55	±	0.53	0.105	NS	0.77	±	0.70	0.74	±	0.04	0.843	NS
Total protein (g/L)	71.67	±	5.17	63.00	±	8.96	0.069	NS	71.33	±	10.04	53.50	±	1.24	0.013	SIG
Albumin (g/L)	35.33	±	2.87	32.67	±	3.79	0.015	SIG	35.00	±	6.57	27.50	±	1.24	0.027	SIG
Globulin (g/L)	36.33	±	3.79	30.33	±	5.74	0.102	NS	36.33	±	3.79	26.00	±	N/A	0.007	SIG
Alb/Glob ratio	0.93	±	0.14	1.07	±	0.14	0.184	NS	0.97	±	0.14	1.05	±	0.12	0.038	SIG
Total Bilirubin	2.33	±	1.43	2.00	±	N/A	0.423	NS	5.33	±	10.04	2.00	±	N/A	0.289	NS
ALK (IU/L)	102.00	±	31.13	102.67	±	21.70	0.958	NS	121.67	±	47.02	109.50	±	16.15	0.387	NS
ALT (IU/L)	50.00	±	2.48	39.00	±	14.90	0.062	NS	45.00	±	21.22	135.50	±	232.27	0.249	NS
AST (IU/L)	101.00	±	33.42	88.33	±	33.73	0.201	NS	88.67	±	19.92	312.00	±	474.47	0.191	NS

NS = not significant; SIG = significant; N/A = not applicable or the test could not be performed; LDL = low-density lipoprotein; Alb/Glob = albumin/globulin; ALK = anaplastic lymphoma kinase; ALT = alanine aminotransferase; AST = aspartate aminotransferase.

**Table 3:** Student’s *t*-test comparing baseline to the end-of-study results at various levels of toxicity for phase one repeat: 14-day acute toxicity study results.

Toxicity Test	Control Baseline			Control End			*p*-Value	Decision	50 mg/kg Baseline			50 mg/kg End			*p*-Value	Decision
Urea	6.90	±	1.79	6.23	±	0.72	0.171	NS	17.07	±	31.43	7.37	±	1.74	0.334	NS
LDL Cholesterol	N/A			N/A				N/A	N/A			N/A				N/A
Triglyceride	0.98	±	0.39	1.09	±	0.60	0.594	NS	0.91	±	0.64	0.88	±	0.93	0.939	NS
Total protein (g/L)	65.67	±	6.25	61.00	±	4.30	0.020	SIG	68.33	±	1.43	59.33	±	3.79	0.016	SIG
Albumin (g/L)	34.33	±	3.79	32.33	±	2.87	0.074	NS	34.33	±	3.79	31.33	±	1.43	0.122	NS
Globulin (g/L)	31.33	±	2.87	28.67	±	1.43	0.015	SIG	34.00	±	2.48	28.00	±	2.48		N/A
Alb/Glob ratio	1.10	±	N/A	1.10	±	N/A		N/A	1.00	±	0.25	1.10	±	N/A	0.225	NS
Total Bilirubin	7.00	±	N/A	9.33	±	3.79	0.118	NS	4.00	±	25.41	3.67	±	7.17	0.500	NS
ALK (IU/L)	189.33	±	106.63	144.67	±	33.08	0.121	NS	170.33	±	55.40	121.67	±	39.85	0.084	NS
ALT (IU/L)	39.33	±	1.43	37.00	±	4.30	0.118	NS	42.00	±	21.66	41.33	±	11.74	0.866	NS
AST (IU/L)	96.67	±	21.13	84.33	±	19.92	0.011	SIG	122.67	±	22.27	139.67	±	231.24	0.792	NS
**Toxicity Test**	**300 mg/kg Baseline**			**300 mg/kg End**			***p*-Value**	**Decision**	**2000 mg/kg Baseline**			**2000 mg/kg End**			***p*-Value**	**Decision**
Urea	11.23	±	12.82	7.83	±	1.60	0.325	NS	6.13	±	5.09	7.47	±	1.12	0.307	NS
LDL Cholesterol	N/A			N/A				N/A	N/A			N/A				N/A
Triglyceride	0.87	±	0.37	1.19	±	1.09	0.317	NS	0.88	±	0.25	0.83	±	0.64	0.768	NS
Total protein (g/L)	67.67	±	5.17	61.00	±	8.96	0.179	NS	67.33	±	3.79	59.67	±	3.79	0.013	SIG
Albumin (g/L)	35.33	±	1.43	32.67	±	1.43	0.057	NS	35.00	±	2.48	31.00	±	2.48	0.020	SIG
Globulin (g/L)	32.33	±	3.79	28.33	±	7.59	0.270	NS	32.33	±	1.43	28.67	±	1.43	0.008	SIG
Alb/Glob ratio	1.10	±	N/A	1.17	±	0.29	0.423	NS	1.10	±	N/A	1.10	±	N/A		N/A
Total Bilirubin	6.33	±	9.40	8.50	±	6.35		N/A	5.67	±	7.99	5.67	±	7.99		N/A
ALK (IU/L)	167.00	±	52.58	124.67	±	77.22	0.061	NS	151.67	±	11.47	132.67	±	19.30	0.027	SIG
ALT (IU/L)	38.67	±	2.87	34.00	±	8.61	0.118	NS	38.33	±	7.99	41.33	±	13.68	0.483	NS
AST (IU/L)	99.33	±	29.43	105.00	±	43.03	0.598	NS	86.67	±	29.64	80.00	±	8.61	0.511	NS

NS = not significant; SIG = significant; N/A = not applicable or the test could not be performed; LDL = low-density lipoprotein; Alb/Glob = albumin/globulin; ALK = anaplastic lymphoma kinase; ALT = alanine aminotransferase; AST = aspartate aminotransferase.

**Table 4:** Student’s t-test comparing baseline to the end-of-study biochemical effects following the administration of various doses of methanolic extracts of *C. flanaganii* stem bark in a subacute toxicity study using Wistar rats.

Variable	100 mg/kg			500 mg/kg			1000 mg/kg			Control		
	Baseline	End	*p*-Value	Baseline	End	*p*-Value	Baseline	End	*p*-Value	Baseline	End	*p*-Value
Urea	6.08 ± 0.62	7.74 ± 2.21	NS	6.47 ± 0.80	9.25 ± 0.56	SIG	6.14 ± 0.61	8.15 ± 2.37	NS	6.26 ± 0.44	9.14 ± 0.73	SIG
LDL Cholesterol	0.15 ± 0.00	0.14 ± 0.03	NS	0.15 ± 0.00	0.15 ± 0.00	N/A	0.15 ± 0.00	0.17 ± 0.03	NS	0.15 ± 0.00	0.15 ± 0.00	N/A
Triglyceride	1.00 ± 0.16	1.06 ± 0.37	NS	0.96 ± 0.17	1.27 ± 0.26	NS	1.09 ± 0.20	0.99 ± 0.34	NS	1.17 ± 0.22	1.31 ± 0.43	NS
Total Protein	59.80 ± 2.65	53.10 ± 13.44	NS	59.90 ± 4.00	60.70 ± 2.31	NS	59.70 ± 4.33	54.60 ± 13.89	NS	59.40 ± 3.54	59.10 ± 1.67	NS
Albumin	32.20 ± 1.71	28.50 ± 7.30	NS	32.40 ± 2.27	32.60 ± 2.08	NS	32.20 ± 2.82	32.33 ± 1.63	NS	31.70 ± 2.16	30.90 ± 1.37	NS
Globulin	21.60 ± 1.02	24.60 ± 6.22	NS	27.50 ± 1.92	28.10 ± 2.17	NS	27.50 ± 1.69	25.50 ± 6.54	NS	27.70 ± 1.47	28.20 ± 1.21	NS
Alb/Glob ratio	1.18 ± 0.03	1.04 ± 0.27	NS	1.19 ± 0.05	1.17 ± 0.14	NS	1.18 ± 0.07	1.04 ± 0.27	NS	1.15 ± 0.04	1.10 ± 0.08	NS
Total Bilirubin	4.30 ± 2.50	4.50 ± 2.72	NS	3.80 ± 1.87	4.70 ± 2.82	NS	2.50 ± 1.92	2.70 ± 2.41	NS	2.50 ± 1.73	2.70 ± 1.55	NS
ALK	255.20 ± 72.65	136.70 ± 51.53	SIG	252.40 ± 86.03	143.40 ± 46.25	SIG	239.10 ± 61.87	136.80 ± 49.24	SIG	288.50 ± 58.88	193.40 ± 45.39	SIG
AST	100.50 ± 7.81	111.90 ± 37.80	NS	96.30 ± 11.06	81.40 ± 9.82	NS	92.00 ± 7.80	78.70 ± 24.08	NS	83.20 ± 6.89	86.60 ± 14.42	NS

NS = not significant; SIG = significant; N/A = not applicable or the test could not be performed; LDL = low-density lipoprotein; Alb/Glob = albumin/globulin; ALK = anaplastic lymphoma kinase; ALT = alanine aminotransferase; AST = aspartate aminotransferase.

**Table 5:** Single-factor ANOVA analysis of subacute chemistry using end-of-study concentration values.

Variable	100 mg/g	500 mg/kg	1000 mg/kg	Control	*p*-Value	Decision
Urea	7.74 ± 2.24	9.25 ± 0.56	8.15 ± 2.37	9.14 ± 0.73	0.412	NS
LDL Cholesterol	0.14 ± 0.03	0.15 ± 0.00	0.17 ± 0.03	0.15 ± 0.00	0.279	NS
Triglyceride	1.06 ± 0.37	1.27 ± 0.26	0.99 ± 0.34	1.31 ± 0.43	0.427	NS
Total Protein	53.10 ± 13.44	60.70 ± 2.31	54.60 ± 13.89	59.10 ± 1.67	0.560	NS
Albumin	28.50 ± 7.30	32.50 ± 2.08	32.33 ± 1.63	30.90 ± 1.37	0.611	NS
Globulin	24.60 ± 6.22	28.10 ± 2.17	25.50 ± 6.54	28.20 ± 1.21	0.513	NS
Alb/Glob ratio	1.04 ± 0.27	1.17 ± 0.14	1.04 ± 0.27	1.10 ± 0.08	0.709	NS
Total Bilirubin	4.50 ± 2.72	4.70 ± 2.82	2.70 ± 2.41	2.70 ± 1.55	0.382	NS
ALK	136.70 ± 51.53	143.40 ± 46.25	136.80 ± 49.24	193.40 ± 45.39	0.194	NS
AST	111.90 ± 37.80	81.40 ± 9.82	78.70 ± 24.08	86.60 ± 14.42	0.125	NS

NS = not significant; LDL = low-density lipoprotein; Alb/Glob = albumin/globulin; ALK = anaplastic lymphoma kinase; ALT = alanine aminotransferase; AST = aspartate aminotransferase.

**Table 6:** Student’s *t*-test comparing baseline to the end-of-study full blood count effects following the administration of various doses of methanolic extracts of *C. flanaganii* stem bark in a subacute toxicity study using Wistar rats.

Variable	100 mg/kg			500 mg/kg			1000 mg/kg			Control		
	Baseline	End	*p*-Value	Baseline	End	*p*-Value	Baseline	End	*p*-Value	Baseline	End	*p*-Value
Red Cell Count	4.23 ± 2.65	5.92 ± 2.24	NS	4.89 ± 2.48	6.53 ± 1.67	NS	6.12 ± 1.59	7.30 ± 0.17	NS	5.69 ± 2.16	7.42 ± 0.16	NS
Haemoglobin	8.69 ± 5.41	11.47 ± 4.35	NS	12.53 ± 4.51	13.94 ± 0.56	NS	14.02 ± 0.66	12.85 ± 3.27	NS	11.72 ± 4.43	14.39 ± 0.27	NS
Haematocrit	0.30 ± 0.19	0.39 ± 0.15	NS	0.34 ± 0.17	0.43 ± 0.11	NS	0.43 ± 0.11	0.44 ± 0.11	NS	0.40 ± 0.15	0.95 ± 1.02	NS
MCV	42.50 ± 26.22	52.70 ± 19.87	NS	54.22 ± 23.67	60.10 ± 15.14	NS	63.90 ± 16.19	60.20 ± 15.22	NS	56.50 ± 21.38	66.80 ± 1.30	NS
MCH	12.40 ± 7.65	15.50 ± 5.85	NS	14.30 ± 7.07	17.40 ± 4.40	NS	20.67 ± 0.54	17.70 ± 4.47	NS	16.50 ± 6.25	19.50 ± 0.51	NS
MCHC	14.70 ± 9.38	18.50 ± 7.77	NS	17.80 ± 9.22	22.20 ± 6.67	NS	25.11 ± 3.55	22.10 ± 6.45	NS	19.80 ± 8.08	24.30 ± 3.89	NS
RDW	8.06 ± 5.06	9.70 ± 3.70	NS	9.44 ± 4.74	11.18 ± 2.85	NS	11.05 ± 3.82	11.51 ± 2.95	NS	9.91 ± 4.65	11.80 ± 0.46	NS
White cell count	3.51 ± 2.28	5.26 ± 2.23	NS	4.50 ± 2.06	6.77 ± 2.28	SIG	4.70 ± 1.31	3.59 ± 1.78	NS	4.30 ± 1.69	5.92 ± 1.07	NS
Neutrophils	5.15 ± 5.47	7.14 ± 4.99	NS	5.00 ± 5.61	6.25 ± 4.67	NS	9.80 ± 8.94	11.37 ± 10.78	NS	8.17 ± 6.77	9.24 ± 7.73	NS
Lymphocytes ABS	25.65 ± 27.15	43.03 ± 30.00	NS	26.56 ± 27.76	36.37 ± 28.96	NS	31.92 ± 26.32	29.96 ± 25.56	NS	33.52 ± 28.15	41.72 ± 28.33	NS
Monocytes ABS	0.07 ± 0.04	0.11 ± 0.06	NS	0.07 ± 0.04	0.16 ± 0.60	SIG	0.08 ± 0.03	0.11 ± 0.06	NS	0.08 ± 0.03	0.16 ± 0.05	SIG
Eosinophils	0.03 ± 0.02	0.06 ± 0.03	NS	0.04 ± 0.03	0.10 ± 0.07	NS	0.05 ± 0.02	0.03 ± 0.02	NS	0.05 ± 0.03	0.06 ± 0.03	NS
Basophils ABS	0.01 ± 0.01	0.01 ± 0.01	NS	0.01 ± 0.00	0.01 ± 0.01	NS	0.01 ± 0.00	0.03 ± 0.03	NS	0.01 ± 0.00	0.01 ± 0.01	NS
Platelet count	385.80 ± 257.90	583.10 ± 231.32	NS	600.30 ± 314.00	745.80 ± 203.92	NS	670.20 ± 185.37	780.00 ± 227.54	NS	674.40 ± 285.65	848.30 ± 90.65	NS

NS = not significant; SIG = significant; MCV = mean corpuscular volume; MCH = mean corpuscular haemoglobin; MCHC = mean corpuscular haemoglobin concentration lymphocytes; ABS = lymphocyte absolute count; Monocytes ABS = monocyte absolute count; Basophils ABS = basophil absolute count.

**Table 7:** Single-factor ANOVA analysis of full blood count using end-of-study concentration values.

Variable	100 mg/g	500 mg/kg	1000 mg/kg	Control	*p*-Value	Decision
Red Cell Count	5.92 ± 2.24	6.53 ± 1.67	7.30 ± 0.17	7.42 ± 0.16	0.535	NS
Haemoglobin	11.47 ± 4.35	13.94 ± 0.56	12.85 ± 3.27	14.39 ± 0.27	0.531	NS
Haematocrit	0.39 ± 0.15	0.43 ± 0.11	0.44 ± 0.11	0.95 ± 1.02	0.287	NS
MCV	52.70 ± 19.87	60.10 ± 15.14	60.20 ± 15.22	66.80 ± 1.30	0.506	NS
MCH	15.50 ± 5.85	17.40 ± 4.40	17.70 ± 4.47	19.50 ± 0.51	0.533	NS
MCHC	18.50 ± 7.77	22.20 ± 6.67	22.10 ± 6.45	24.30 ± 3.89	0.538	NS
RDW	9.70 ± 3.70	11.18 ± 2.85	11.51 ± 2.95	11.80 ± 0.46	0.631	NS
White cell count	5.26 ± 2.23	6.77 ± 2.28	3.59 ± 1.78	5.92 ± 1.07	0.070	NS
Neutrophils	7.14 ± 4.99	6.25 ± 4.67	11.37 ± 10.78	9.24 ± 7.73	0.796	NS
Lymphocytes ABS	43.03 ± 30.00	36.37 ± 28.96	29.96 ± 25.56	41.72 ± 28.33	0.8777	NS
Monocytes ABS	0.11 ± 0.06	0.16 ± 0.06	0.11 ± 0.06	0.16 ± 0.5	0.375	NS
Eosinophils	0.06 ± 0.03	0.10 ± 0.07	0.03 ± 0.02	0.06 ± 0.03	0.088	NS
Basophils ABS	0.01 ± 0.01	0.01 ± 0.01	0.03 ± 0.03	0.01 ± 0.01	0.539	NS
Platelet count	583.10 ± 231.32	745.80 ± 203.92	780.00 ± 227.54	848.30 ± 90.65	0.190	NS

NS = not significant; SIG = significant; MCV = mean corpuscular volume; MCH = mean corpuscular haemoglobin; MCHC = mean corpuscular haemoglobin concentration; Lymphocytes ABS = lymphocyte absolute count; Monocytes ABS = monocyte absolute count; Basophils ABS = basophil absolute count.

**Table 8:** The Shapiro–Wilk test for normality.

Organ	W Statistic	Degrees of Freedom (df)	*p*-Value
Liver	0.931	37	0.023
Spleen	0.948	37	0.081
Kidney	0.913	37	0.007

## Data Availability

All supporting data are attached along with the article.

## Supplementary Materials

The following supporting information can be downloaded on figshare at: https://figshare.com/s/9406c96e44b711864afe (accessed on 10 June 2023). File S1: The ARRIVE Guidelines Author Checklist.
